# *Arabidopsis thaliana* growth is independently controlled by the SUMO E3 ligase SIZ1 and Hexokinase 1

**DOI:** 10.17912/micropub.biology.000270

**Published:** 2020-06-29

**Authors:** Pedro Humberto Castro, Nuno Verde, Herlander Azevedo

**Affiliations:** 1 CIBIO, InBIO - Research Network in Biodiversity and Evolutionary Biology, Universidade do Porto, Campus Agrário de Vairão, 4485-661 Vairão, Portugal; 2 BioSystems & Integrative Sciences Institute (BioISI), Plant Functional Biology Center, University of Minho, Campus de Gualtar, 4710-057 Braga, Portugal; 3 Departamento de Biologia, Faculdade de Ciências, Universidade do Porto, Rua Campo Alegre, 4169-007 Porto, Portugal

**Figure 1:**
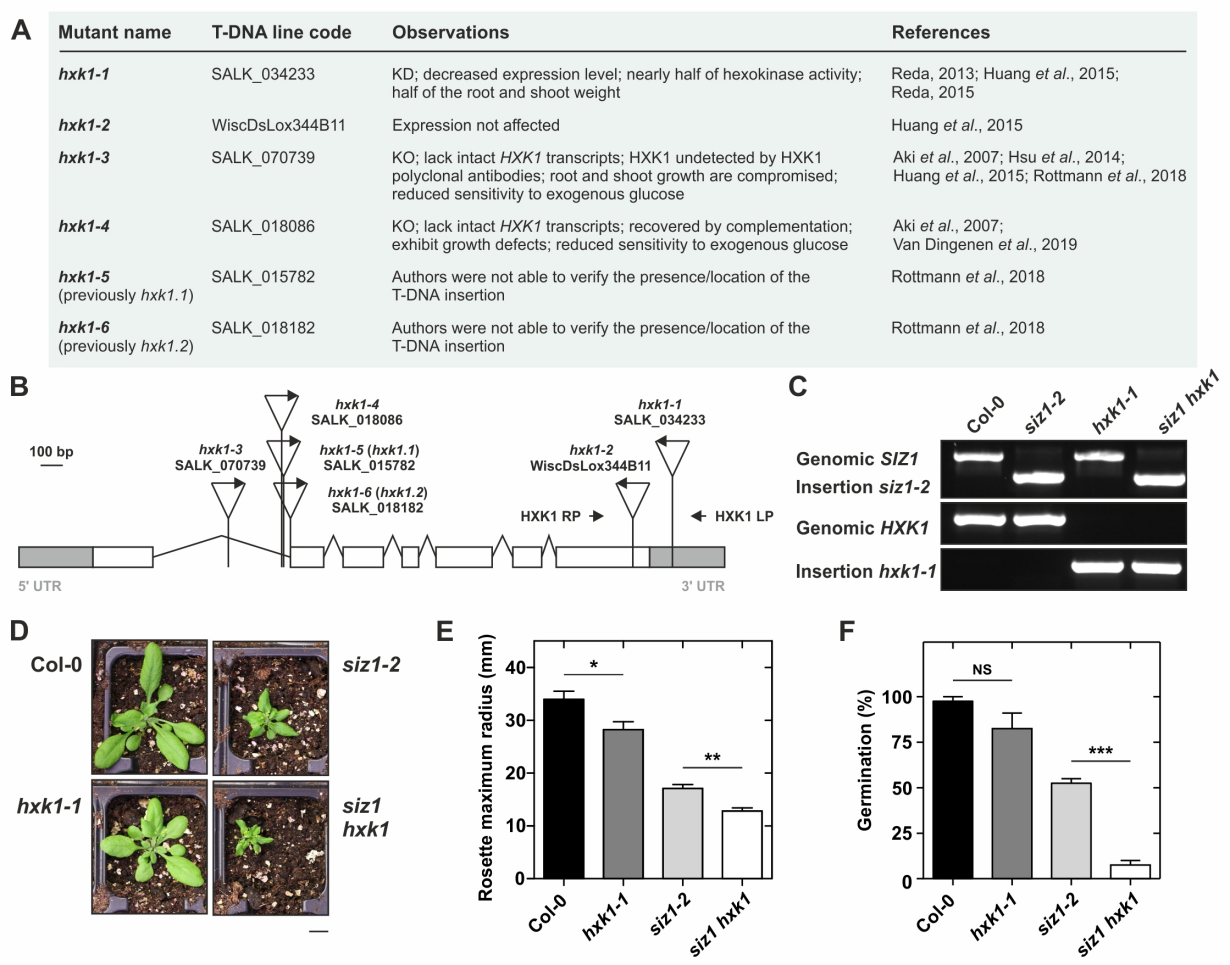
Characterization of the double T-DNA insertion mutant *siz1-2 kxk1-1* (*siz1 hxk1*). (**A**) Summarized information regarding *HXK1* T-DNA insertion mutants in the *Arabidopsis thaliana* Columbia-0 (Col-0) ecotype background, with updated designations; KD and KO stand for knockdown and knockout, respectively. (**B**) Representation of the *HXK1* gene displaying exons (white boxes), introns (thin lines), and UTRs (grey boxes). The site and orientation of the T-DNA insertion (triangle with insertion mutant line code) and location of primers used for genotyping are represented; scale bar indicates 100 base pairs (bp). (**C**) Genotyping PCR, confirming the presence or absence of the T-DNA insertion for the *hxk1-1* and *siz1-2* mutant lines. (**D**) Morphology of 1-month-old plants grown under long days. Scale bar indicates 1 cm. (**E**) Morphological measurement of the maximum rosette radius for each genotype. Error bars represent SEM (n≥5). (**F**) Seed germination percentages scored by green cotyledon appearance 10 days after sowing onto MS media; error bars represent SEM (n≥4). Asterisks represent statistically significant differences between genotypes (unpaired t test; NS, non-significant; *, P < 0.05; **, P < 0.01; ***, P < 0.001).

## Description

Carbohydrate metabolism needs to be tightly regulated in order to ensure an organism’s survival. For this reason, several sensing mechanisms are in permanent alert to monitor and maintain homeostatic levels of sugars (Li and Sheen, 2016). For instance, glucose cellular levels can be sensed by different signaling proteins, including the Hexokinase 1 (HXK1), a moonlighting protein with both metabolic (glucose phosphorylation) and sensing/transduction functions (Moore *et al.*, 2003). In a situation of excess glucose, HXK1 forms a nuclear complex that directly inhibits photosynthesis-related genes, reducing the synthesis of more glucose (Cho *et al.*, 2006). Interestingly, HXK1 loss-of-function mutants lose the capacity to sense external sugar and, consequently, are capable of germinating in high percentages of external sugar supplementation. Indeed, the reference HXK1 mutant *gin2-1*, that harbors a nonsense mutation (Q432*) in *Arabidopsis thaliana* ecotype Landsberg *erecta* (L*er*), was obtained from a forward screening for *glucose insensitive* (*gin*) mutants that do not suffer post-germinative growth arrest (PGGA) on a 6% glucose MS medium (Moore *et al.*, 2003). Broadly, a myriad of phenotypic defects have been described for HXK1 mutants, highlighting the protein’s importance in the regulation of plant development (Aguilera-Alvarado and Sanchez-Nieto, 2017; Van Dingenen *et al.*, 2019). Considering HXK1’s importance to plant fitness and development, one expects its activity to be tightly controlled.

During a previous study (Castro *et al.*, 2015), we aimed to link carbohydrate signaling with the post-translational modification by the small peptide Small Ubiquitin-like Modifier (SUMO). SUMO conjugation is a reversible and rapid modification that can regulate the activity, localization and stability of target proteins (Castro *et al.*, 2012). The covalent attachment of SUMO to a target (i.e. sumoylation), is mediated by an enzymatic cascade that sequentially involves DSP proteases (for SUMO maturation), E1 activating enzymes, E2 conjugating enzymes, and E3 ligases (Castro *et al.*, 2012; Castro *et al.*, 2018a). SIZ1 is an E3 ligase that is critical to enhance SUMO conjugation, especially in response to environmental stressors. Accordantly, *SIZ1* loss-of-function mutants are not only developmentally compromised, but also hypersensitive to several environmental stress conditions (Castro *et al.*, 2012).

Previously, we reported that the *siz1* mutation results in sugar hypersensitivity in its osmotic stress component, and also in the signaling effect of sugars leading up to PGGA (Castro *et al.*, 2015; Castro *et al.*, 2018b). Moreover, *siz1* presents less glucose, fructose, sucrose and starch, which is likely correlated with enhanced expression of sugar degradation genes (Park *et al.*, 2012; Tomanov *et al.*, 2014; Castro *et al.*, 2015). Interestingly, the glucose sensor transcript *HXK1* and HXK-metabolic pathway marker genes were differentially expressed in *siz1*, and for this reason, we aimed to test for the presence of epistatic relationships between SIZ1 and HXK1. Consequently, we crossed *siz1-2* with the previously used *HXK1* T-DNA insertion mutant SALK_034233 (Reda, 2013; Huang *et al.*, 2015; Reda, 2015), hereafter designated *hxk1-1* (Fig. 1A,B). Since the *siz1-2* mutant is a SALK line in the Columbia-0 (Col-0) background, we also opted for a SALK mutant for *HXK1*, instead of the well-characterized *gin2-1* (in L*er* background). During our inspection for Col-based insertion mutant lines present in the literature, we came across conflicting mutant allele nomenclatures. As such, in Fig. 1A,B we try to summarize existing knowledge, and suggest a novel nomenclature for several *HXK1* insertion mutant lines for the community. In the F2 generation, *hxk1-1* plants were genotyped using the primers displayed in Fig. 1B, resulting in the identification of the double mutant *siz1-2 hxk1-1* (*siz1 hxk1*) (Fig. 1C). The *hxk1-1* mutant was smaller than the wild-type, similar to other *hxk1* alleles (Reda, 2013; Huang *et al.*, 2015; Reda, 2015; Van Dingenen *et al.*, 2019). Most significantly, the double mutant *siz1 hxk1* showed greater morphological defects than *siz1-2* (Fig. 1D), which suggests that *hxk1-1* might produce an additive effect to the dwarfism displayed by the *siz1-2* mutant (Fig. 1E). The germination also seems additively affected in the double mutant with only a few seeds germinating and producing cotyledons (Fig. 1F). In comparison with other *hxk1* alleles, *hxk1-1* presents a milder developmental defect (Aki *et al.*, 2007; Huang *et al.*, 2015; Van Dingenen *et al.*, 2019). Since the *hxk1-1* T-DNA insertion is in the 3’UTR (Fig. 1B) it is most likely a knockdown mutant allele. In support, Huang *et al.* (2015) showed a slight decrease of *HXK1* expression in the *hxk1-1* line. Albeit these results suggest that *hxk1-1* is not a complete loss-of-function mutant, Reda (2013, 2015) stated that it lacks *HXK1* expression (not shown by the author) and showed that this same mutant line is compromised in hexokinase activity by ~45%. This *HXK1* knockdown mutant may be useful for genetic strategies where *hxk* mutants are introgressed into mutant backgrounds that are also severely pleiotropic, as is the current case of *siz1*. Nevertheless, the epistatic relationship between *HXK1* and *SIZ1* should be further clarified using null alleles of *HXK1*.

Collectively, these results support that SIZ1 and HXK1 are likely to be involved in independent growth control pathways. This also reinforces the idea that the HXK1-signaling pathway is unlikely to involve SIZ1 (Castro *et al.*, 2015). However, the possible involvement of SUMO in the control of HXK1 activity, especially its nuclear localization and regulation of gene expression, cannot be discarded. This is supported by the presence of evolutionarily conserved sumoylation motifs in this protein family (Castro *et al.*, 2020). Moreover, HXK1 was detected in a yeast-two-hybrid screening for SUMO E2 conjugase enzyme (SCE) interacting partners, and was sumoylated in a heterologous bacterial system (Elrouby and Coupland, 2010), which may indicate the existence of pathways alternative to SIZ1. This would mean either direct SUMO modification by E2 conjugases, or the action of alternative E3 ligases, in the modulation of HXK1 activities.

## Methods

**Plant handling:** *Arabidopsis thaliana* mutant lines are in the ecotype Columbia-0 background. The T-DNA insertion mutants *siz1-2* (SALK_065397) and *hxk1-1* (SALK_034233) were ordered from the NASC European Arabidopsis Stock Centre (arabidopsis.info) and confirmed by genotyping using the following primers: LBb1.3 5’-ATTTTGCCGATTTCGGAAC-3’; SIZ1-2 RP 5’-CACGACAGATGAAGCATTGTG-3’; SIZ1-2 LP 5’-GAGCTGAAGCATCTGGTTTTG-3’; HXK1 RP 5’-AGCTCGTCTCTCTGCTGCTGGA-3’; HXK1 LP 5’-CAGAACTCCAGTGAAGTGAGCTTTGA-3’. Synchronized Arabidopsis seeds were stratified, surface sterilized and sown onto MS medium as previously described by Castro *et al.* (2015). Plants were grown in growth chambers with a 16 h light/8 h dark cycle under cool white light (80 µE m^-2^s^-1 ^light intensity) at 22-24ºC. For standard growth, in-vitro-grown 7-day-old seedlings were transferred to a soil to vermiculite (4:1) mixture and maintained with regular watering.

**Phenotyping:** Rosette size was measured using the ImageJ software (https://imagej.nih.gov/ij/). Germination percentages were determined 10 days after plating onto half-strength MS medium, by scoring green cotyledon appearance using a stereomicroscope.
